# Harnessing SmartPhones to Personalize Nutrition in a Time of Global Pandemic

**DOI:** 10.3390/nu13020422

**Published:** 2021-01-28

**Authors:** Niv Zmora, Eran Elinav

**Affiliations:** 1 Immunology Department, Weizmann Institute of Science, Rehovot 7610001, Israel; niv.zmora@weizmann.ac.il; 2 The Sackler Faculty of Medicine, Tel-Aviv University, Tel Aviv 6997801, Israel; 3 The Research Center for Digestive Tract and Liver Diseases, Tel Aviv Sourasky Medical Center, Tel Aviv 6423906, Israel; 4 Division of Cancer-Microbiome Research, DKFZ, 69120 Heidelberg, Germany

**Keywords:** Nutrition app, smartphone, nutrition, COVID-19

## Abstract

The soar in COVID-19 cases around the globe has forced many to adapt to social distancing and self-isolation. In order to reduce contact with healthcare facilities and other patients, the CDC has advocated the use of telemedicine, i.e., electronic information and telecommunication technology. While these changes may disrupt normal behaviors and routines and induce anxiety, resulting in decreased vigilance to healthy diet and physical activity and reluctance to seek medical attention, they may just as well be circumvented using modern technology. Indeed, as the beginning of the pandemic a plethora of alternatives to conventional physical interactions were introduced. In this Perspective, we portray the role of SmartPhone applications (apps) in monitoring healthy nutrition, from their basic functionality as food diaries required for simple decision-making and nutritional interventions, through more advanced purposes, such as multi-dimensional data-mining and development of machine learning algorithms. Finally, we will delineate the emerging field of personalized nutrition and introduce pioneering technologies and concepts yet to be incorporated in SmartPhone-based dietary surveillance.

## 1. The Pandemic Information Age

SmartPhone ownership is estimated at 45% of the world’s population for 2020, and this figure is only expected to increase over the next years, especially in developing nations. As mobile devices are prevalent, carried around by users throughout their daily lives and offer various sensing modalities, they are compelling targets of data collection, monitoring and interaction, allowing not only for facilitating user feedback, but also for offering accessible custom-made interventions.

The COVID-19 pandemic is unique in the sense that it employed SmartPhone-based technologies for epidemiological purposes. Population surveillance and screening for the disease were carried out in the United States through an app that collected SmartWatch and activity tracker data, as well as surveys for self-reported symptoms [1,2]. As such, a study conducted on large cohorts from the United States and the United Kingdom, based on self-reporting of symptoms through a SmartPhone app, revealed that loss of sense of smell and taste could be included as part of routine screening for COVID-19 [3,4]. In China, mobile phone geolocation data ware used to map the distribution of confirmed cases to assist in decision-making to slow the rate of transmission [5]. Similarly, contacts of infected users were tracked using GPS data or Bluetooth (short-range radio) signals [6,7].

Additionally, SmartPhone-based technology and auxiliary plug-in devices can assist in case identification. Viral RNA can be detected through point-of-care testing using a portable assay and a SmartPhone-based reader [8]. Voice analysis is an emerging field that strives to detect specific vocal ‘biomarkers’, such as shortness of breath and cough, associated with COVID-19 from recordings obtained by mobile devices [9].

More digital technologies developed to diagnose or curb COVID-19 spread are extensively covered elsewhere [10].

## 2. The Advent of SmartPhone Apps in Dietary Surveillance

Nutrition apps provide a simple way for the user to log meals and other daily activities, obtain nutritional information on food items and receive automatic push notifications based on preset thresholds and conditions. They allow dietitians and other medical personnel to assess the users’ compliance and diet transgressions, track their behaviors, set goals, send motivational messages, and analyze the data. These features rendered these apps an attractive means of providing remote dietary care to users, irrespective of their COVID-19 status. Herein, we will highlight the various usages of nutrition apps, and then focus on special considerations pertaining to the COVID-19 pandemic.

The last decade has witnessed a surge in nutrition apps, rendering SmartPhones a validated dietary assessment tool [11,12]. Several studies have suggested that users were more compliant with dietary tracking using Smartphone apps compared to the traditional paper-and-pencil food diaries [13], although these findings were inconsistent [14]. Furthermore, a recent international survey completed by healthcare professionals (including dietitians, doctors and nurses) revealed that 45.5% recommended nutrition apps to their patients [15].

Nutrition apps have been widely used to promote dietary interventions, such as increasing fruit and vegetable consumption or decreasing saturated fat and sugar-sweetened beverage intake [16,17,18,19]. They have also served as a tool in the armamentarium to prevent or combat obesity in children [20], adolescents [21], and adults with specific medical conditions, such as pregnancy [22] and type 2 diabetes mellitus (T2DM) [23]. With regards to the latter, a simple intervention of sending text messages providing information, motivation, support and reminders related to diabetes management improved glycemic control in poorly controlled individuals with T2DM [24]. Nutrition apps have been used in research for other indications, including maintaining a low-salt diet in patients suffering from cardiovascular disease [25], optimizing food choices for patients suffering from nephrolithiasis [26], and monitoring energy intake and nutritional behaviors in elite athletes [27,28]. It should be noted, however, that many of the studies that showed promising results for SmartPhone-based strategies were limited by short-term intervention periods and were not sufficiently methodologically rigorous.

The call for social distancing and self-isolation during the COVID-19 pandemic has promoted a surge in SmartPhone apps as alternatives to social interactions. Various services, including retail businesses and healthcare have adapted to the changing reality with telecommunication. Likewise, diet and nutrition advice is facing a burning need to shift from physical clinical encounters to remote interactions. Patients with COVID-19, especially the elderly, people of low socioeconomic and educational status or those with underlying medical conditions require continuous dietary care, presenting more complex and challenging problems for healthcare professionals, including increased catabolism, nutritional deficiencies or altered physiology [29,30,31,32]. Even individuals without COVID-19, who suffer from chronic diseases or malnutrition, may require adaptations to reduce food insecurity during the pandemic [33]. Furthermore, healthy individuals who have experienced lifestyle changes and become sedentary due to imposed lockdowns may need dietary modifications, and others may benefit from remote nutritional counseling to counteract psychological distress due to the health emergency situation [34,35]. In the following sections we will illustrate the role of nutrition SmartPhone apps as means to tackle these issues.

## 3. Requirements of Nutrition Apps

**Figure 1:**
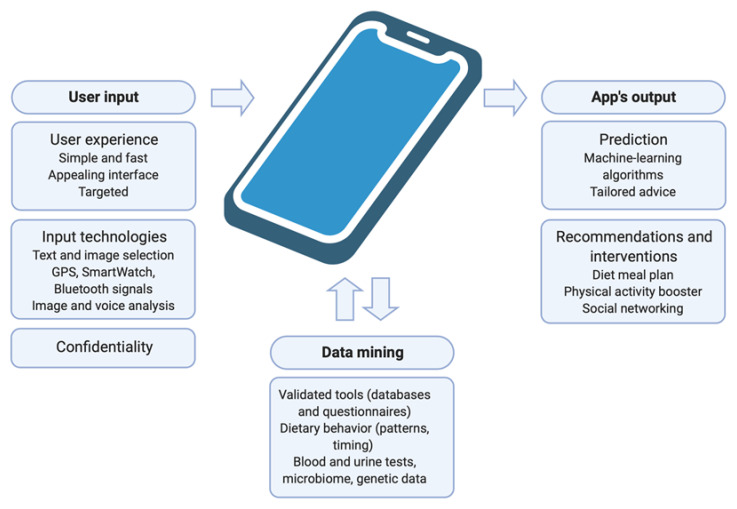
Requirements of nutrition apps. SmartPhone apps consist of several modules: the interface should provide compelling user experience and utilize advanced technologies to facilitate comprehensive data collection, and users’ confidentiality should be maintained. Data mining should be based both on entries recorded by the app and externally derived data and should be suitable for population-based studies. Data analysis should be performed using artificial intelligence and subsequent recommendations should be produced at a personal level (Created with BioRender.com).

Generating an effective nutrition SmartPhone app is a task that requires numerous considerations and encompasses a multi-disciplinary effort and a collaboration of product managers, software engineers, medical personnel, scientists and data analysts. Apps should comply with a standardized Nutrition Care Process (NCP), and therefore follow the framework of nutrition assessment, diagnosis, intervention, monitoring and evaluation to ensure efficient and reliable remote continuous care [36]. All apps should offer an expeditious and simplified means to log or report meal intake, and many implement additional features of planning, reminding, coaching, boosting motivation and providing information (Figure 1).

User experience is of paramount importance. As users adherence tends to abate over time [37] and is directly correlated with the app’s efficacy [38], great emphasis should be placed on its target audience, for instance, while a vivid interface may appeal to children, elderly users may require enhanced accessibility. The design should be attractive, enjoyable and easy-to-use, and navigation should be swift and seamless. The most prevalent recording methods used on mobile phone platforms are electronic food diaries (assisted by textual or picture-based databases), 24-h recall, barcode scanners and food photograph analysis. Employing image analysis technologies in nutrition apps as an alternative to written records has considerably evolved over the last years. Initially, carbohydrate estimation required lengthy redrawing of food items [39]. Subsequently, artificial intelligence allowed for calorie, carbohydrate or macronutrient estimation of meal images, which outperformed or was non-inferior to evaluation by experienced dietitians [40,41]. Image analysis technologies can be stand-alone or complimented by a voice recording to explain the contents of the photograph and then analyzed by a dietitian [42]. Novel technologies offer food image recognition with increasing accuracy harnessing deep neural network architectures [43].

Food databases should not only be comprehensive and up-to-date, but also customized to fit the users’ locale. As such, tables of food composition should be country-specific to allow for reliable and accurate reporting of meals. Additionally, neonates, inpatients or individuals suffering from chronic medical conditions should be able to log nutritional formulas and food supplements. Decision-making should rely on validated methods, for instance a recent study proposed a remote nutritional screening tool during the COVID-19 pandemic based on the Malnutrition Universal Screening Tool (MUST) and the SARC-F questionnaire [44]. Finally, user confidentiality and data protection principles must be ensured in every nutrition app, as stipulated by the General Data Protection Regulation (GDPR). Namely, data reported by users should be collected and stored in an anonymized (or pseudonymized) manner, allowing full traceability while ensuring participant protection. Meticulous security audit, including penetration tests and vulnerability assessments should be conducted by the app developers to prevent data leaks. Data must not be shared or transmitted to third-party companies without informed consent, which should disclose all data sharing practices. Additionally, the Children’s Online Privacy Protection Act (COPPA) should be enforced in nutrition apps addressed to minors.

## 4. Scientific Applications of Nutrition Apps and Their Pitfalls

Data collected by nutrition apps are usually processed by individuals and their healthcare providers to track their compliance with a dietary regimen and assess the effect of nutrition interventions. Nonetheless, this information, when acquired adequately on large populations, can serve as real-world data, a complementary source to randomized control trials to reveal statistically significant health trends and yield robust and meaningful conclusions in nutritional management. These studies are particularly crucial in times of changing social and economical behaviors, such as the COVID-19 pandemic. Furthermore, nutritional data interlaced with blood tests results deemed potentially valuable and warrants further research in patients with COVID-19, as circumstantial evidence linked between deficiencies in some dietary constituents and disease severity [45,46].

As previously implied, albeit effective in increasing “attentive eating” as well as promoting specific dietary modifications among users affected by medical conditions (e.g., reducing salt intake [47]), nutrition apps are often not more valid or reliable than conventional methods [48], and do not always translate into clinical benefit (e.g., cardiovascular risk reduction [49], weight loss [50] or prevention of gestational diabetes mellitus [51]), posing marginal added value compared to standard counseling on diet and physical activity [49]. These discouraging findings merit reevaluation of universal dietary recommendations and call for a more individualized approach when designing nutrition interventions.

## 5. Practicing Personalized Diet with Nutrition Apps

**Figure 2:**
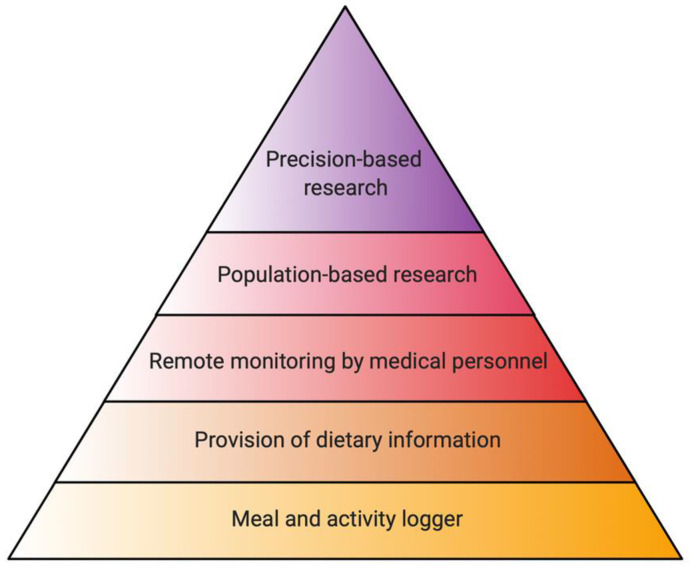
-Strata of nutrition apps functionality. The foundation of every nutrition SmartPhone app rests on a user interface to log meals and daily activities. Most apps provide visualization of the recorded data and information to the user. More advanced features include direct supervision and remote monitoring by medical personnel. High-level operations on collected data are population- and precision-based studies (Created with BioRender.com).

A large body of evidence has demonstrated that there is great variability in responses to similar food among different individuals [52]. This variability derives from a multitude of factors, including personal parameters as well as the consumer’s environmental milieu. Therefore, optimal dietary planning should be made in the context of an individual and their unique features, rendering the notion of “one-diet-fits-all” obsolete (Figure 2). Host-intrinsic variability stems from genetic and epigenetic factors. As such, methylation of adipogenic genes correlated with insulin resistance after a specific nutritional intervention [53].

Environmental factors that affect responses to dietary interventions include eating patterns, timing of meals and their relation to physical activities and to psychological stress [54]. These factors, termed dietary behavior, can be modeled based on SmartPhone data and dictate interventions [55], as advanced sensing capabilities embedded in mobile devices allow for estimation of daily energy expenditure from phone accelometry and elucidating time-location eating patterns using GPS data [55]. Machine learning techniques employed on these data may assist in tracing and predicting dietary lapses during dietary interventions, and have been shown to result in greater precision when applied on individually segregated data, rather than data pooled from a group of individuals [55,56].

Of the many factors shaping individualized responses to food the gastrointestinal microbiome has been gaining increasing attention. The microbiome, constituting trillions of bacteria and other microorganisms, has a major role in food digestion and exerts a profound metabolic effect on the host. Studies have found that certain microbiome features might account for altered responses to a particular dietary intervention, for instance a high ratio between two bacterial genera, *Prevotella* and *Bacteroides*, in stool was associated with greater weight loss [57] and improved glucose metabolism [58].

Our lab has profiled a large cohort of individuals in terms of anthropometrics, blood tests and stool microbiome composition and function, and tracked their blood glucose levels with continuous glucose monitors for a period of one week, while they were instructed to keep a food and activity diary through a designated SmartPhone app. We found, like others, a high inter-individual variability in post-meal glucose responses to the same food. We then associated these differences with individual features and constructed a decision tree-based machine-learning algorithm to predict post-meal glucose responses. Eventually, we conducted a small-scale interventional study to prove that individualized menus devised based on our algorithm could effectively maintain normoglycemia [59]. Similarly, a recent study proposed using a SmartPhone application to manage gestational diabetes mellitus in the time of COVID-19. The app incorporated diet transgressions, blood glucose values and ketonuria to make adjustment recommendations regarding diet and insulin treatment [60]. In summary, personalized nutrition is a holistic multi-faceted concept, which relies on various host, microbiome and environmental variables. The widespread use of nutrition SmartPhone apps complemented by biomarkers and advanced multi-omics analyses set the groundwork for exploring, practicing and fine-tuning personalization of diet.

## 6. Future Prospects

We envision that the commodification and rapid developments in mobile technology and the recent breakthroughs in big data analysis will result in an upcoming burgeon to the field of personalized nutrition. Incorporating multi-dimensional host and microbiome data profiling with tracking of food intake and food behavior will yield high-quality tailor-made dietary recommendations, which can be optimized for specific objectives or medical conditions.

Studies will further explore determinants of intra-individual differences, such as the addition or omission of micronutrients [61], the effect of prior meals on the response to consequent meals [62] or the effect of the circadian phase on responses to food [63]. With regards to the latter, it has been shown that restriction of daily eating hours was associated with reduced body weight and long-lasting improvement in various symptoms [64].

Ongoing alterations in individual features will require dietary recommendations to be periodically adjusted. This and the aforementioned associations between several micronutrients levels and COVID-19 severity may require frequent blood tests or microbiome profiling, or the development of plug-in assays for point of care quantification of nutrient deficiencies, such as serum iron, vitamin A and vitamin B12 [65,66].

Moreover, SmartPhone apps will transcend beyond provision of nutritional recommendations to provide active behavior modifications, so users will implicitly make healthier choices. An interesting example to this notion was witnessed in users of the mobile app Pokémon GO, who increased their number of steps after installation of the game [67].

Thinking outside the box may aid in improving compliance and instilling motivation to pursue nutritional and lifestyle interventions. For instance, one study proposed an integrative system for adolescents, which consists of garments embedded with monitoring devices, activity trackers, a web portal and a several smartphone apps with reward and a gamification modules [68]. Likewise, interface between nutrition apps and social networking websites may boost adherence in some users.

With these ideas in mind, dietary surveillance and decision-making in this time of global pandemic may pose many challenges; however SmartPhone apps may potentially overcome them and pave the way towards augmented and more effective personalized nutrition.

## Data Availability

Not applicable.
